# Eosinophilic Solid and Cystic Renal Cell Carcinoma—A Systematic Review and Meta-Analysis

**DOI:** 10.3390/ijms25115982

**Published:** 2024-05-30

**Authors:** Andrada Loghin, Maria Cătălina Popelea, Ciprian Doru Todea-Moga, Iuliu Gabriel Cocuz, Angela Borda

**Affiliations:** 1 Histology Department, “George Emil Palade” University of Medicine, Pharmacy, Science and Technology of Targu Mures, 540142 Targu Mures, Romania; andradaloghin@yahoo.com (A.L.); angela.borda@umfst.ro (A.B.); 2 Pathology Department, Mures Clinical County Hospital, 540011 Targu Mures, Romania; iuliu.cocuz@umfst.ro; 3 Urology Department, “George Emil Palade” University of Medicine, Pharmacy, Science and Technology of Targu Mures, 540142 Targu Mures, Romania; ciprian.todea@gmail.com; 4 Urology Department, Mures Clinical County Hospital, 540011 Targu Mures, Romania; 5 Pathophysiology Department, “George Emil Palade” University of Medicine, Pharmacy, Science and Technology of Targu Mures, 540142 Targu Mures, Romania; 6 Department of Pathology, Targu-Mureș Emergency County Hospital, 540139 Targu Mures, Romania

**Keywords:** eosinophilic solid and cystic renal cell carcinoma, ESC-RCC, TSC gene mutation, unclassified renal cell carcinoma, tuberous sclerosis complex, emerging entity

## Abstract

Eosinophilic solid and cystic renal cell carcinoma (ESC-RCC) is a novel and uncommon type of renal cell carcinoma, which has been recently recognized and introduced as a distinct entity in the WHO 2022 kidney tumor classification. Previously known as “unclassified RCC”, followed by “tuberous sclerosis complex (TSC)-associated RCC”, ESC-RCC is now a distinct category of kidney tumor, with its own name, with specific clinical manifestations, and a unique morphological, immunohistochemical and molecular profile. Due to its recent introduction and the limited available data, the diagnosis of ESC-RCC is still a complex challenge, and it is probably frequently misdiagnosed. The secret of diagnosing this tumor lies in the pathologists’ knowledge, and keeping it up to date through research, thereby limiting the use of outdated nomenclature. The aim of our case-based review is to provide a better understanding of this pathology and to enrich the literature with a new case report, which has some particularities compared to the existing cases.

## 1. Introduction

The World Health Organization (WHO) 2022 kidney tumor classification has introduced a novel and uncommon type of renal cell carcinoma (RCC) known as eosinophilic solid and cystic renal cell carcinoma (ESC-RCC) [1]. Due to its recent introduction and the limited description of around 70 cases in the literature, this entity remains a complex challenge and is probably frequently misdiagnosed or underutilized. ESC-RCC is characterized by its indolent biological behavior and low potential for metastasis [2], highlighting the critical importance of distinguishing it from other types of renal tumors. Further investigations are necessary to comprehensively ascertain the biological behavior of ESC-RCC, as there are currently only a limited number of well-documented series.

We present the case of a 58-year-old man with sporadic ESC-RCC located in the left kidney, and summarize the existing literature, presenting clinical, pathological, immunohistochemical and molecular data in order to improve our knowledge of this tumor and minimize diagnostic inaccuracies. To the best of our knowledge, this is the first case reported in the literature from Romania and Eastern Europe.

The aim of our case-based literature review was to emphasize the transition of ESC-RCC from a non-classified RCC to a well-defined entity, assessing all the literature that has discussed this type of tumor and presenting a case of ESC-RCC that was diagnosed in our Pathology Department.

## 2. Results

### 2.1. Case Presentation

We present the case of a 58-year-old man admitted to the Urology Department of Târgu-Mureș Clinical County Hospital, Romania, with macroscopical hematuria and a tumor in the left kidney detected in imaging. The patient had arterial hypertension, with no other significant medical history, and he was in apparently good health at the time of admission. The patient underwent a left laparoscopic radical nephrectomy, and the specimen was sent to the Pathology Department for histopathological examination and final diagnosis. Macroscopically, the kidney exhibited a relatively well-defined, non-encapsulated tumor, with apparent necrotic and hemorrhagic content, measuring 12 cm at its maximum diameter.

**Figure 1:**
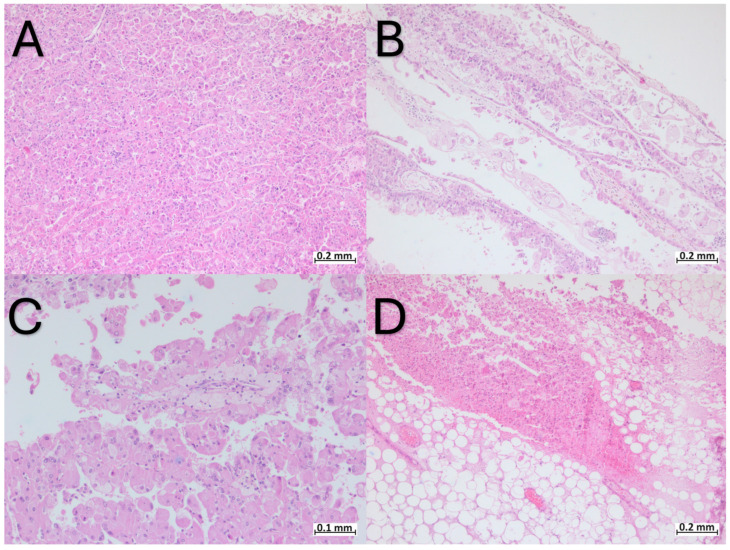
Heterogenous architecture of ESC-RCC: (**A**) solid architecture; (**B**) cystic architecture; (**C**) papillary architecture and foamy macrophages in the core of the papillary structures; (**D**) tumoral cells with areas of necrosis infiltrating the adipose tissue of the hilum.

Microscopically, the tumor had a solid, cystic, and occasionally papillary architecture (Figure 1A–C).

**Figure 2:**
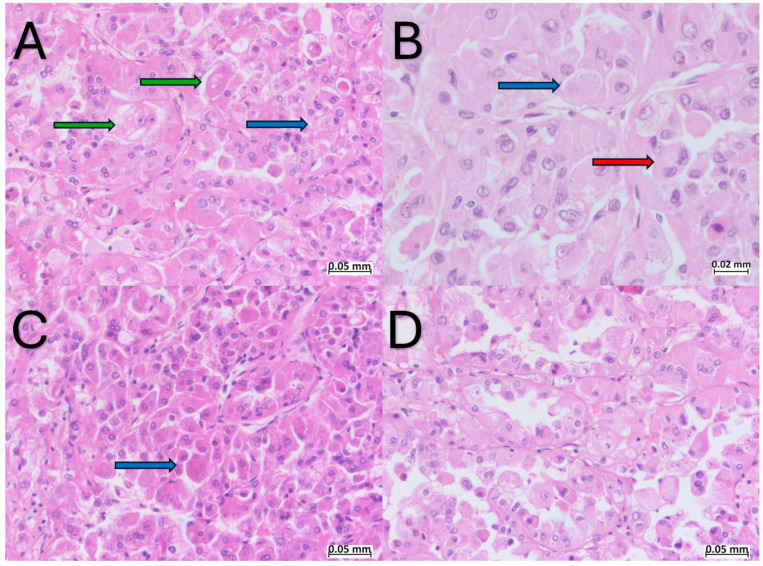
Hematoxylin–Eosin, morphology of ESC-RCC: (**A**–**C**) polygonal cells, with abundant eosinophilic cytoplasm, round to oval nuclei, moderate pleomorphism, and visible nucleoli along cells with small clusters of basophilic granules (“leshmania-like” bodies) within the cytoplasm (blue arrows). Binucleated cells (red arrow) and tumor cells with intracytoplasmic vacuoles (green arrows) are also observed; (**D**) hobnail appearance cells delineating microcystic structures.

The tumor cells were polygonal, with abundant eosinophilic cytoplasm, round to oval nuclei, moderate pleomorphism, and visible nucleoli (Figure 2). Focally, binucleated cells were observed and the tumor cells displayed intracytoplasmic vacuoles and small clusters of basophilic granules (Figure 2). The cystic structures were lined by a single layer of cells with a “hobnail” appearance (Figure 2D). Within the core of the papillary structures, groups of foamy macrophages were noted (Figure 1C). Upon microscopical examination, significant regions of hemorrhage and tumoral necrosis were noted. The tumor infiltrated the adipose tissue in the hilum (Figure 1D), without infiltrating the perirenal adipose tissue. Lymphatic and vascular invasion was not observed. The renal parenchyma adjacent to the tumor showed hyalinized glomeruli and features of thyroidization in the renal tubules. In the hilum, two lymph nodes without metastases were observed.

**Figure 3:**
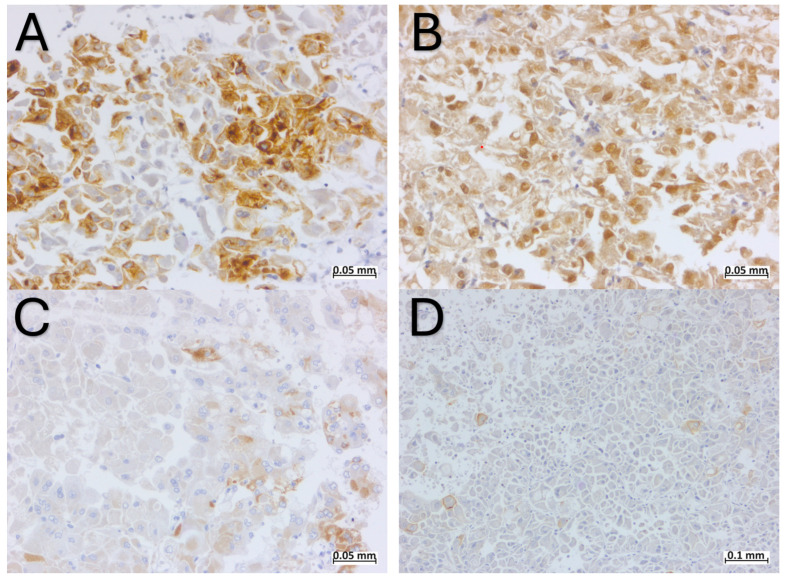
Immunohistochemical profile of ESC-RCC: (**A**) focal positivity for CK 20; (**B**) positivity for PAX 8; (**C**) focal positivity for AMACR; (**D**) focal positivity for CD10.

**Figure 4:**
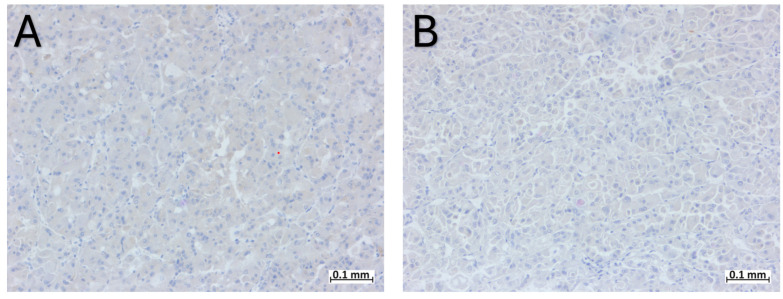
Immunohistochemical profile of ESC-RCC: (**A**) negativity for CK7; (**B**) negativity for CD117.

The tumor cells were positive for PAX8, weakly and focally positive for CK20, CKAE1/AE3, CD10, and AMACR (Figure 3), and negative for CK7, CD117, MelanA, HMB45, TFE3, Actin, and ALK (Figure 4). The expression of FH and SDHB was preserved.

A complementary molecular study was also conducted, revealing a mutation in TSC1 and JAK1, confirming the hypothesis of ESC-RCC. The gene alterations of our case are summarized in Table 1. The final stage of the tumor was pT3a, due to the invasion of the adipose tissue of the hilum.

The case was discussed in the multi-disciplinary team (MDT), and the “watch and wait” strategy was decided by the board members. The patient is well and alive, disease-free, 10 months after surgery.

### 2.2. From Unclassified to a Distinct Type of Tumor in ESC-RCC

In recent years, our understanding of renal tumors has markedly advanced, due to the progress in molecular pathology. Numerous new entities have been identified, and existing tumors have been reclassified following thorough examination of clinical features, morphology, immunohistochemistry, and genetic mutations. Oncocytic renal tumors, previously classified as unclassified, now hold a distinct diagnostic category due to their significant prognostic implications and clinical relevance [3].

Renal cell carcinomas (RCCs) linked with tuberous sclerosis complex (TSC) were first documented in 1996 [4]. Subsequently, three more extensive modern series have been documented, consistently indicating that TSC-associated RCCs manifest at a younger age, predominantly affect females (7:3 ratio), and often present as multifocal or bilateral [4,5,6,7]. In 2014, two groups presented a significant series of TSC-associated RCCs, establishing the foundation for the current classification system. Yang et al. documented 46 tumors in 19 individuals with TSC, categorizing them in the following manner: (1) papillary RCC (52%), (2) hybrid oncocytic chromophobe tumors (33%), and (3) unclassified RCCs (15%) [6]. The second study, conducted by Guo and colleagues, examined 57 RCCs in 18 TSC patients, delineating them into the following: (1) renal angiomyoadenomatous tumor (30%), (2) chromophobe-like renal cell carcinomas (59%), and (3) granular eosinophilic/macrocystic renal cell carcinomas (11%) [7]. Despite variations in nomenclature between these two studies, they documented comparable morphologies and immunohistochemical patterns that led to several publications over the years that characterized similar tumors in sporadic cases with mutations in the TSC/mTOR pathway [8]. Therefore, the concept of ESC-RCC was mentioned even back then as one of the three types of renal tumors associated with TSC, but it was initially identified as “unclassified” in Yang’s study and “granular eosinophilic/macrocystic renal cell carcinomas” in Guo’s study.

Presently, patients diagnosed with TSC exhibit tumors categorized into four main groups: (1) RCC featuring leiomyomatous stroma (RCC-LMS), (2) eosinophilic solid and cystic RCC (ESC-RCC), (3) low-grade oncocytic tumor (LOT), and (4) eosinophilic vacuolated tumor (EVT) [9,10].

Later, in 2016, Trpkov et al. [11] described a distinct, sporadic tumor, with female predilection, indolent progression, and unique histopathological features, showing morphological similarity to those formerly identified as granular eosinophilic macrocystic RCC occurring in patients with TSC. Consequently, Trpkov introduced the term eosinophilic solid and cystic renal cell carcinoma (ESC-RCC) to characterize the tumor they identified, which manifests sporadically in clinical settings among patients lacking a diagnosis of TSC [12]. Since this report, additional cases of sporadic ESC-RCC have been described in different research studies, with an estimated occurrence rate of 0.07% to 0.2% within all RCC [13].

In recent findings, it has been observed that TSC and ESC-RCC rarely occur together, with only about 10% of individuals with ESC-RCC experiencing simultaneous clinical symptoms of TSC [1]. WHO introduced this nomenclature last year, in the newest edition, recognizing this rare pathology as a distinct tumor with distinguishable histopathological characteristics.

### 2.3. Etiology, Epidemiology, and Clinical Manifestations

The exact etiology of ESC-RCC is not fully understood. However, it is known that this tumor was formerly acknowledged to predominantly occur in individuals with tuberous sclerosis complex (TSC) [12]. Tuberous sclerosis complex is a syndrome that affects multiple organs such as the skin, brain, heart, lung, or kidney, with a chance of 2–4% to develop kidney cancer [3,12]. It represents an autosomal dominant disorder, affecting around 2,000,000 people worldwide [14,15]. TSC results from germline loss of function mutations in either TSC1, located on chromosome 9q34, or TSC2, located on chromosome 16p13.3 [14,15,16]. Mutations in TSC2 are present in approximately 70% of TSC patients and generally correlate with a higher disease burden and severity compared to TSC1 mutations [9,16]. The genes TSC1 and TSC 2 constitute a complex together with TBC1D7 that manages the function of mTOR complex 1. The inactivation of these two genes abolishes the downregulation of mTORc1, leading to increased cell growth and oncogenesis; therefore, the dysregulation of this pathway may contribute to the development and progression of ESC-RCC [17].

Initially identified as granular eosinophilic, macrocystic or unclassified RCC, ESC-RCC constitutes approximately 7–11% of all renal epithelial neoplasms associated with TSC [18]; however, most of the cases are sporadic [19,20]. Although specific associations have not been definitively established, certain environmental exposures or lifestyle factors might contribute to the development of ESC-RCC. In general, the development of ESC-RCC likely results from a complex interaction among genetic, molecular, and environmental factors. Additional research is required to completely understand the underlying mechanisms driving the emergence of this uncommon form of renal cell carcinoma.

ESC-RCCs typically present as solitary, small size, and low-stage tumor, with few multifocal and bilateral cases reported in the literature [21,22,23,24] and is predominantly described in women. The onset age has a wide range, including pediatric patients, with an average of 57 years of age [1,4]. The current incidence is unclear, as numerous cases were previously categorized as “unclassified RCCs” or other entities, due to the lack of nomenclature [19]. Clinical symptoms are often absent, and the tumor may be discovered incidentally during imaging tests conducted for reasons unrelated to the tumor [22].

### 2.4. Histopathological, Immunohistochemical and Molecular Pathology

#### 2.4.1. Histopathological Features

The specific morphological characteristics along with the systematically immunohistochemical profile typically provide adequate criteria for diagnosing ESC-RCC.

Macroscopically, ESC-RCC typically presents as a well-defined, non-encapsulated tumor, grossly displaying both solid and cystic growth. The presence of macrocysts is a prominent gross feature, although there are occasional instances where only microscopic cysts are observed [5,25]. The majority of documented tumors exhibited a small size, with a mean of 4.2 cm. However, the reported tumor sizes showed variability, ranging from 1.2 to 13.5 cm in the greatest dimension, as indicated by the largest series reported by Trpkov et al. [26,27].

Microscopically, the tumor is well-defined, without a well-formed fibrous capsule at the periphery. The solid areas display a diffuse, acinar, or nested pattern of growth. The tumoral cells present abundant eosinophilic, granular cytoplasm, containing coarse basophilic granules (referred to as “stippling”, or “leshmania-like” bodies). These granules represent accumulations of rough endoplasmic reticulum, as visualized through electron microscopy [28]. In the cysts, cells with hobnail arrangement could be found. The nuclei are typically round to oval, and while present, nucleoli are generally not prominently visible, comparable to WHO/ISUP grade 2 or 3 [28]. Psammoma bodies, clusters of multinucleated cells, small groups of foamy macrophages and lymphocytes can be seen. Additionally, a focal papillary pattern or other morphological variations such as clear cell features, or tubular growth have been described. Micro- or macro-cystic areas are usually lined by cells with a hobnail appearance [4,5,24]. In a recent study, melanin pigment was identified in one ESC-RCC [29].

#### 2.4.2. Immunohistochemical Features

The most specific immunohistochemical profile of ESC-RCC involves positive staining for Cytokeratin 20 (CK20) and negative staining for Cytokeratin 7 (CK7). While ESC-RCC may exhibit weak CK7 positivity, and in approximately 10–15% of cases [30], CK20 may be negative [28], a scenario where CK7 is intense and diffuse positive and CK20 is negative rules out the possibility of an ESC-RCC diagnosis. The immunohistochemical profile of ESC-RCC also includes positive staining for CK AE1/AE3, CK 8/18 [4], Vimentin, AMACR, and PAX 8 and negativity for CD117, CD10, and CA IX [1,4,25,28]. Sharma et al. reported one case with focal CD117 positivity, in an area with oncocytoma-like features [28]. In our case, there was focal positivity for CD10, a discovery that aligns with the study conducted by Trpkov et al. [11], where CD10 was focally positive in 77% of the cases. The positivity of melanocytic markers (Cathepsin-K, HMB45, and Melan-A) is a distinctive discovery within ESC-RCC. It appears that Cathepsin-K and Melan-A expressions are more commonly observed compared to HMB45, as reported in previous studies [5,30]. The complete immunohistochemical profile of ESC-RCC is shown in Table 2.

#### 2.4.3. Molecular Features

The molecular analysis of sporadic ESC-RCC using next-generation sequencing (NGS) has revealed somatic bi-allelic mutations in the TSC genes, specifically TSC1 and TSC2 in the great majority of cases [4,24,31,32,33]. In the context of sporadic cases, ESC-RCC exhibits a predilection for women, and is associated with the biallelic loss or mutations altering the TSC1 or TSC2 gene [34,35]. In a study conducted by Mehra et al., six out of seven cases of sporadic ESC-RCC presented biallelic loss of TSC1 or TSC2, leading to the conclusion that TSC mutations have significance in the genesis of ESC-RCC [36]. In their studies, Palsgrove et al. and Munari et al. also found the presence of TSC1 or TSC2 mutations in 100% of the sporadic ESC-RCC cases [37,38]. ESC-RCC carries mutations in TSC1 or TSC2, with recurrent mutually exclusive somatic bi-allelic loss of TSC1/2, being recognized as a molecular marker for ESC-RCC [25,29,39]. The absence of germline TSC abnormalities in matched non-neoplastic renal parenchyma serves as a distinguishing factor between ESC-RCC and its syndromic counterpart (TSC-RCC) [40]. In their study, Munari et al. [6] conducted multi-regional tumor sampling using NGS on ESC-RCC. They established the uniform presence of TSC1 mutation across all analyzed tumor samples, confirming its clonal origin. This finding offers additional support for the consistent occurrence of TSC gene dysfunction in ESC-RCC. Palsgrove et al. have additionally verified the consistent occurrence of mutations in either the TSC1 or TSC2 genes in pediatric ESC RCC (88% of cases) and adult ESC-RCC (100% of cases). This observation encompasses a metastatic ESC-RCC case that exhibited a full response to mTOR-targeted therapy [24,28]. Mutations in the TSC1 and TSC2 genes inhibit the mTOR complex, resulting in the activation of mTORC1 and the subsequent dysregulation of downstream cellular pathways involved in the proliferation and growth of cells. The molecular karyotype analysis of ESC-RCC conducted by Trpkov et al. has revealed frequent and recurrent genomic alterations. Their findings indicated that the tumor may demonstrate copy number amplification of chromosomes 16, 7, 13q, and 19p, as well as copy number deletion of Xp11.21 and 22q11.23, along with loss of heterozygosity of TSC1 and TSC2 [23].

While these molecular changes are not exclusive or specific to ESC-RCC, when considered alongside the morphological and immunohistochemical characteristics unique to ESC-RCC, they indicate a relatively well-defined morpho-molecular entity [28,41].

### 2.5. Differential Diagnosis

Considering the varying therapeutic approaches and prognostic implications associated with different renal tumors, it is important to distinguish ESC-RCC from other RCCs. This distinction is particularly critical due to the distinct therapeutic strategies and prognosis specific to each type of renal tumor, thereby ensuring optimal patient management.

Firstly, a comprehensive range of renal tumors featuring eosinophilic cells should be taken into consideration in the differential diagnosis of ESC-RCC. This category of RCC includes renal oncoytoma (RO), the eosinophilic variant of chromophobe renal cell carcinoma (Chr-RCC), and some less common entities like succinate dehydrogenase (SDH)-deficient RCC, MiTF translocation RCC (especially TFEB), and epithelioid angiomyolipoma (AML) [28].

Secondly, TSC-associated RCCs are categorized into four primary tumor types: ESC-RCC, RCC with leiomyomatous stroma (RCC-LMS), low-grade oncocytic tumor (LOT), and eosinophilic vacuolated tumor (EVT). Therefore, the differential diagnosis of ESC-RCC should be made with the other TSC-associated RCCs. Each of the three newly identified renal tumors (ESC-RCC, LOT, and EVT) exhibits a distinctive morphology, relatively uniform immunoprofiles, and specific molecular genetic characteristics. While LOT and EVT are oncocytic tumors, with abundant, prominent mitochondria in the cytoplasm, ESC-RCC is an eosinophilic tumor, with abundant eosinophilic cytoplasm, but not oncocytic cytoplasm [40]. As its name suggests, EVT (formerly known as high-grade oncocytic tumor—HOT) displays a high-grade morphology, abundant eosinophilic cytoplasm, and prominent intracytoplasmic vacuoles, being easily distinguishable from ESC-RCC. In rare instances of ESC-RCC, sporadic vacuoles may be observed, but thick-walled vessels (characteristic for EVT) are typically absent. While immunohistochemistry can provide some guidance, it is worth noting that ESC-RCC may occasionally test negative for CK20, whereas EVT may rarely exhibit CK20 positivity in individual cells [40]. LOT is characterized by cell uniformity and the CK7+/CD117− immunophenotype, while ESC-RCC lacks cell uniformity. In this instance, the primary basis for the differential diagnosis relies on morphological characteristics, given their similar immunophenotype. It is noteworthy that ESC-RCC, EVT, and LOT can coexist within the same kidney, which is not unexpected due to the common genetic drivers underlying all three tumors. Such a remarkable discovery was recorded in a patient with TSC [16]. RCC-LMS is characterized by a smooth muscle stroma (Actin positive), which separates the tumoral cells (CK7 positive) into well-defined tumor nodules [10]. Anaplastic lymphoma kinase rearrangement-associated renal cell carcinoma (ALK-RCC) should be taken into consideration for a differential diagnosis due to the gross features (solid and cystic appearance) and due to the heterogenous microscopical aspect, with varied architecture and cytomorphology, including solid and cystic architecture and tumoral cells with eosinophilic cytoplasm and intracytoplasmic vacuoles.

However, the specific morphology of ESC-RCC, especially the solid and cystic architecture, abundant eosinophilic cytoplasm, and intracytoplasmic basophilic granules, combined with the specific immunohistochemical profile (CK20+/CK7−), should be sufficient for accurately establishing the diagnosis.

In Table 3, we summarized the main morphological differences between ESC-RCC and renal tumors with oncocytic/eosinophilic cells while the main differential diagnosis regarding immunophenotype is presented in Table 4.

### 2.6. Treatment and Prognosis

The gold standard for treating ESC-RCC remains surgical resection through either partial or radical nephrectomy, depending on the stage of the tumor [1]. ESC-RCC carries mutations in TSC1 or TSC2, with recurrent mutually exclusive somatic bi-allelic loss of TSC1/2, resulting in hyperactive mTORC1 signaling. Recently, Tjota et al. [35] highlighted that neoplasms associated with TSC/MTOR form a distinct group with diverse morphology, IHC staining, and clinical behavior. Fortunately, there are currently mTOR inhibitors available for the treatment of RCCs [42]. Prior to recent times, all cases of ESC-RCC were exclusively documented in adult female patients, typically presenting low-stage, unilateral tumors, with favorable prognoses, with no evidence of recurrence or metastases [11,23]. Sakhadeo et al. [30] conducted a study comprising a case series, where two out of three patients, both females, presented with metastatic disease. One case showed liver metastases, while the other exhibited para-aortic lymphnode and bone metastases. These findings contribute to the growing evidence of the aggressive nature and metastatic propensity of these tumors. At present, the estimated metastasis rate for ESC-RCC ranges from 3% to 5% [43]; however, the precise prognosis and metastatic potential remain to be conclusively determined, as only a limited number of reports have documented metastases [5,21]. A study conducted by the designation of “renal cell carcinoma” for this entity is justified by these documented rare cases of ESC-RCC with metastatic disease, emphasizing the need for ongoing clinical follow-up and surveillance in these patients [5,24].

## 3. Discussion

Eosinophilic solid and cystic renal cell carcinoma is a new entity, recently introduced in the WHO classification of renal tumors, released in 2022. This tumor, with all its morphological characteristics, has been known under various names such as “unclassified”, “granular eosinophilic carcinoma” or “eosinophilic macrocystic carcinoma”. The name of ESC-RCC was first mentioned in 2016 by Trpkov et al. [11], and it was thought to be a TSC-associated RCC. Later, ESC-RCC was described in non-syndromic patients, arriving at the present moment, when it is known that around 90% of the cases are sporadic. However, the loss of function of TSC1/TSC2 genes are very frequent in the sporadic ESC-RCC; hence, ESC-RCC is thought to be potentially indicative of TSC gene mutations [10].

The patient presented in this article was known to have arterial hypertension, with no other clinical manifestation of TSC. Although our patient was not diagnosed with TSC syndrome, the molecular study that was conducted revealed a mutation of the TSC1 gene. This finding corresponds to the data in the literature, confirming that most cases of ESC-RCC are sporadic and that ESC-RCC is a good predictor for the presence of TSC1/TSC2 mutations. ESC-RCC is described mostly in female patients, with variable onset ages, including pediatric patients, with an average age of 57 years of age. Grossly, the tumor size is also variable, being mostly small, under 5 cm, with most tumors being diagnosed at stage pT1 (under 4 cm). In our case, the patient was a 58-year-old male, with a solitary tumor measuring 12 cm at its maximum diameter. The tumor was also infiltrating the adipose tissue of the hilum, resulting in a pT3a stage. These epidemiological and macroscopical features represent the particularity of the case portrayed in the occurrence of ESC-RCC in a middle-aged man, accompanied by an unusually large size and advanced TNM stage. In terms of clinical manifestations, ESC-RCC is described as an asymptomatic disease, with no obvious signs or symptoms, and it is usually discovered incidentally [41]. In our case, the patient presented at the Urology Department with macroscopical hematuria as a solitary and principal symptom. The classical morphology described in the literature was also found in our case, with solid and cystic architecture, abundant granular eosinophilic cytoplasm with basophilic granules, and cysts lined by hobnail cells. The uniqueness of our case lies in the presence of extensive areas of hemorrhage and necrosis, along with the tumoral infiltration of the adipose tissue of the hilum.

The immunohistochemical profile of the present case was specific for ESC-RCC, with strong and diffuse positivity for PAX8 and focal positivity for CK20, CD10 and AMACR. Although there was only focal positivity for CK20, together with the negativity for CK7, CD117, and melanocytic markers, brought us one step closer to the final diagnosis. In our case, there were no differences among the immunohistochemical features described in the literature, once again proving the importance of immunohistochemistry in pathology.

The molecular study, which demonstrated a TSC1 mutation, confirmed the hypothesis of our presumptive diagnosis of ESC-RCC. Therefore, we can conclude that there is a strong association between ESC-RCC and the presence of TSC1/2 gene mutations, even though the molecular characteristics of this particular type of renal cell carcinoma require further investigation.

## 4. Materials and Methods

We have performed a case-based literature review by assessing all the literature that has described the ESC-RCC both before and after the new classification. The review is structured in a narrative manner, assessing the literature that was identified in PubMed and Web of Science databases, starting from the oldest article, which was found in 1996, until today. A total number of 44 articles were found and included in the study (original research, case presentations, and reviews). Our literature study was structured such that, due to the limited number of available studies, no additional criteria were established for including articles beyond the specified keywords: eosinophilic solid and cystic renal cell carcinoma, unclassified renal cell carcinoma, granular eosinophilic renal cell carcinoma, tuberous sclerosis complex, emerging entity, and new entity. Besides those articles, a few articles that were relevant to our research were included in the study.

Related to our literature findings, we attached to our literature review a presentation of a case of ESC-RCC diagnosed in the Mures Clinical County Hospital of Targu Mures Romania and Hospices Civils de Lyon, France. Informed consent was obtained from the patient, and the study was approved by the ethics committee of the hospital (Protocol code 3924/12 April 2024). A basic histological and immunohistochemistry assessment was performed in the Mures Clinical County Hospital, and molecular pathology was conducted in Hospices Civils de Lyon, France.

## 5. Conclusions

In conclusion, ESC-RCC is a rare tumor with a unique morphology, immunohistochemical profile, and molecular characteristics, which has been clearly defined as an entity in the new WHO 2022 classification. In this article, we summarized the available data and knowledge regarding ESC-RCC and presented a case from our Pathology Department, with the aim of contributing to the medical literature and providing a significant addition to the understanding of this rare condition. The particularity of the case presented in this case-based review lies in the occurrence of ESC-RCC in a middle-aged man, accompanied by an unusually large size of the tumor, the presence of extensive areas of necrosis and hemorrhage, and an advanced TNM stage. We hope that the number of ESC-RCC diagnoses will increase alongside the publication of novel studies that will help pathologists to enhance their knowledge regarding ESC-RCC.

## Figures and Tables

**Table 1:** Pathogenic gene alteration.

Gene	Reference Sequence	Exon	Variant	Variant Protein Alteration	Depth (X)	Sensitivity (%)
TSC1	NM_000368	15	c.1598del	p.(Pro533Leufs*23)	1148	0.87
JAK1	NM_001320923	16	c.1988- 91_2005dup	p.(Phe669*)	897	1.11

**Table 2:** Immunohistochemical profile of ESC-RCC.

IHC Marker	Staining
CK20	+
CK7	−/+ (focal)
CKAE1/AE3	+/−
CK8/18	+
Vimentin	+
AMACR	+
PAX8	+
CD117	−/+ (focal)
CD10	−/+ (focal)
CA IX	−
Cathepsin-K	−/+
HMB45	−/+
Melan-A	−/+

**Table 3:** Differential diagnosis of ESC-RCC regarding morphological aspects.

	Morphological Features
ESC-RCC	Solid and cystic architecture, abundant eosinophilic cytoplasm with basophilic granules; cysts lined by hobnail cells
RO	Solid, nested architecture; oncocytic cells, granular eosinophilic cytoplasm
Chr-RCC	Eosinophilic cells, wrinkled nuclei, perinuclear halo
SDHB-deficient RCC	Eosinophilic, clumpy cytoplasm, intracytoplasmic vacuoles
MiTF-RCC	Two-cell population: large with eosinophilic/pale cytoplasm and small eosinophilic cells
E-AML	Polygonal epithelioid cells, eosinophilic cytoplasm, large nuclei with prominent nucleoli
LOT	Uniform cells, round nuclei, prominent nucleoli, perinuclear halo; edematous areas with solitary cells
EVT	High-grade appearance, large nucleoli, oncocytic cells with prominent intracytoplasmic vacuoles

ESC-RCC: eosinophilic solid and cystic renal cell carcinoma; RO: renal oncocytoma; Chr-RCC: chromophobe carcinoma; SDHB-deficient RCC: succinate dehydrogenase subunit B deficient renal cell carcinoma; MiTF-RCC: microphthalmia-associated transcription factor renal cell carcinoma; E-AML: epithelioid angiomyolipoma; LOT: low-grade oncocytic tumor; EVT: eosinophilic vacuolated tumor.

**Table 4:** Differential diagnosis by immunophenotype.

	CK20	CK7	CD117	PAX8	CD10	AE1/AE3	Vim	MelanA	HMB45	SDHB
ESC-RCC	+	−/+	−	+	−/+	+/−	+	−/+	−/+	+
RO	−	−	+	+	−	+	−	−	−	+
Chr-RCC	−	+	+	+	−	+	−	−	−	+
SDHB-deficient RCC	−	−	−	+	−	+/−	−	−	−	−
MiTF	−	−	−	+	+	−	+	+/−	+/−	+
E-AML	−	−	−	−	−	−	+	+	+	+
LOT	−	+	−	+	−	+	−	−	−	+
EVT	−	−	+	+	+	+	−	−	−	+

ESC-RCC: eosinophilic solid and cystic renal cell carcinoma; RO: renal oncocytoma; Chr-RCC: chromophobe carcinoma; SDHB-deficient RCC: succinate dehydrogenase subunit B deficient renal cell carcinoma; MiTF-RCC: microphthalmia-associated transcription factor renal cell carcinoma; E-AML: epithelioid angiomyolipoma; LOT: low-grade oncocytic tumor; EVT: eosinophilic vacuolated tumor.

## Data Availability

Data is contained within the article.

## References

[1] He X., Chen Y., Tang H., Xu Y., Zhu X., Wang C., Chen Q., Guo D. (2023). Eosinophilic Solid and Cystic Renal Cell Carcinoma with TSC2 Mutation: A Case Report and Literature Review. Diagn. Pathol..

[2] Wang L., Jiang J. (2021). Eosinophilic Solid and Cystic Renal Cell Carcinoma: A New Entity. Asian J. Surg..

[3] Sharma R., Thirunavukkarasu B., Elhence P., Rodha M.S., Sureka B. (2022). Eosinophilic Solid and Cystic Renal Cell Carcinoma: From Unclassified to Classified, a Case Report. Turk. J. Pathol..

[4] Dibble C.C., Cantley L.C. (2015). Regulation of MTORC1 by PI3K Signaling. Trends Cell Biol..

[5] Tretiakova M.S. (2018). Eosinophilic Solid and Cystic Renal Cell Carcinoma Mimicking Epithelioid Angiomyolipoma: Series of 4 Primary Tumors and 2 Metastases. Hum. Pathol..

[6] Munari E., Settanni G., Caliò A., Segala D., Lonardi S., Sandrini S., Vacca P., Tumino N., Marconi M., Brunelli M. (2022). TSC Loss Is a Clonal Event in Eosinophilic Solid and Cystic Renal Cell Carcinoma: A Multiregional Tumor Sampling Study. Mod. Pathol..

[7] Mehra R., Vats P., Cao X., Su F., Lee N.D., Lonigro R.J., Premkumar K., Trpkov K., McKenney J.K., Dhanasekaran S.M. (2018). Somatic Bi-Allelic Loss of TSC Genes in Eosinophilic Solid and Cystic Renal Cell Carcinoma. Eur. Urol..

[8] Guo J., Tretiakova M., Troxell M.L., Osunkoya A.O., Fadare O., Sangoi A.R., Shen S.S., Lopez-Beltran A., Mehra R., Heider A. (2014). Tuberous Sclerosis–Associated Renal Cell Carcinoma. Am. J. Surg. Pathol..

[9] Gupta S., Stanton M.L., Reynolds J.P., Whaley R.D., Herrera-Hernandez L., Jimenez R.E., Cheville J.C. (2022). Lessons from Histopathologic Examination of Nephrectomy Specimens in Patients with Tuberous Sclerosis Complex: Cysts, Angiomyolipomas, and Renal Cell Carcinoma. Hum. Pathol..

[10] Henske E.P., Cornejo K.M., Wu C.-L. (2021). Renal Cell Carcinoma in Tuberous Sclerosis Complex. Genes.

[11] Trpkov K., Hes O., Bonert M., Lopez J.I., Bonsib S.M., Nesi G., Comperat E., Sibony M., Berney D.M., Martinek P. (2016). Eosinophilic, Solid, and Cystic Renal Cell Carcinoma. Am. J. Surg. Pathol..

[12] Mohaghegh Poor S.M., Mathur S., Kassier K., Rossouw J., Wightman R., Saranchuk J., Gibson I.W. (2021). Two Cases of Sporadic Eosinophilic Solid and Cystic Renal Cell Carcinoma in Manitoba Population. Int. J. Surg. Pathol..

[13] Xu H., Li Z., Cui F., Zhang Y., Yan D., Zhang Y. (2022). Imaging Features of Eosinophilic Solid and Cystic Renal Cell Carcinoma: An Additional Case Report of a Novel Tumor Entity. Urol. Case Rep..

[14] Northrup H., Aronow M.E., Bebin E.M., Bissler J., Darling T.N., de Vries P.J., Frost M.D., Fuchs Z., Gosnell E.S., Gupta N. (2021). Updated International Tuberous Sclerosis Complex Diagnostic Criteria and Surveillance and Management Recommendations. Pediatr. Neurol..

[15] Gupta S., Jimenez R.E., Herrera-Hernandez L., Lohse C.M., Thompson R.H., Boorjian S.A., Leibovich B.C., Cheville J.C. (2021). Renal Neoplasia in Tuberous Sclerosis: A Study of 41 Patients. Mayo Clin. Proc..

[16] Lerma L.A., Schade G.R., Tretiakova M.S. (2021). Co-Existence of ESC-RCC, EVT, and LOT as Synchronous and Metachronous Tumors in Six Patients with Multifocal Neoplasia but without Clinical Features of Tuberous Sclerosis Complex. Hum. Pathol..

[17] Dibble C.C., Elis W., Menon S., Qin W., Klekota J., Asara J.M., Finan P.M., Kwiatkowski D.J., Murphy L.O., Manning B.D. (2012). TBC1D7 Is a Third Subunit of the TSC1-TSC2 Complex Upstream of MTORC1. Mol. Cell.

[18] Machacek M.E., Wu C.-L., Cornejo K.M. (2024). Pathology of Hereditary Renal Cell Carcinoma Syndromes: Tuberous Sclerosis Complex (TSC). Semin. Diagn. Pathol..

[19] Trpkov K., Williamson S.R., Gill A.J., Adeniran A.J., Agaimy A., Alaghehbandan R., Amin M.B., Argani P., Chen Y.-B., Cheng L. (2021). Novel, Emerging and Provisional Renal Entities: The Genitourinary Pathology Society (GUPS) Update on Renal Neoplasia. Mod. Pathol..

[20] Tjota M.Y., Pankhuri W., Segal J.P., Antic T. (2021). TSC/MTOR-Mutated Eosinophilic Renal Tumors Are a Distinct Entity That Is CK7+/CK20-/Vimentin-: A Validation Study. Hum. Pathol..

[21] Li Y., Reuter V.E., Matoso A., Netto G.J., Epstein J.I., Argani P. (2017). Re-Evaluation of 33 “Unclassified” Eosinophilic Renal Cell Carcinomas in Young Patients. Histopathology.

[22] Alomar K., Alia L., Safaa Q., Fadel M., Orabi A.A., Alhussein A.A. (2023). A Rare Case of Eosinophilic Solid and Cystic Renal Cell Carcinoma in a 48-Year-Old Woman: Case Report and Literature Review. Int. J. Surg. Case Rep..

[23] Trpkov K., Abou-Ouf H., Hes O., Lopez J.I., Nesi G., Comperat E., Sibony M., Osunkoya A.O., Zhou M., Gokden N. (2017). Eosinophilic Solid and Cystic Renal Cell Carcinoma (ESC RCC): Further Morphologic and Molecular Characterization of ESC RCC as a Distinct Entity. Am. J. Surg. Pathol..

[24] Palsgrove D.N., Li Y., Pratilas C.A., Lin M.-T., Pallavajjalla A., Gocke C., De Marzo A.M., Matoso A., Netto G.J., Epstein J.I. (2018). Eosinophilic Solid and Cystic (ESC) Renal Cell Carcinomas Harbor TSC Mutations: Molecular Analysis Supports an Expanding Clinicopathologic Spectrum. Am. J. Surg. Pathol..

[25] Parilla M., Kadri S., Patil S.A., Ritterhouse L., Segal J., Henriksen K.J., Antic T. (2018). Are Sporadic Eosinophilic Solid and Cystic Renal Cell Carcinomas Characterized by Somatic Tuberous Sclerosis Gene Mutations?. Am. J. Surg. Pathol..

[26] Perrino C.M., Grignon D.J., Williamson S.R., Idrees M.T., Eble J.N., Cheng L. (2017). Morphological Spectrum of Renal Cell Carcinoma, Unclassified: An Analysis of 136 Cases. Histopathology.

[27] Andeen N.K., Qu X., Antic T., Tykodi S.S., Fang M., Tretiakova M.S. (2018). Clinical Utility of Chromosome Genomic Array Testing for Unclassified and Advanced-Stage Renal Cell Carcinomas. Arch. Pathol. Lab. Med..

[28] Siadat F., Trpkov K. (2020). ESC, ALK, HOT and LOT: Three Letter Acronyms of Emerging Renal Entities Knocking on the Door of the WHO Classification. Cancers.

[29] Aldera A.P., Hes O. (2021). Eosinophilic Solid and Cystic Renal Cell Carcinoma with Melanin Pigment—Expanding the Morphological Spectrum. Int. J. Surg. Pathol..

[30] Desai S., Sakhadeo U., Yadav S., Bakshi G., Prakash G., Katdare A., Menon S. (2021). Eosinophilic Solid Cystic Renal Cell Carcinoma: A Series of 3 Cases Elucidating the Spectrum of Morphological and Clinical Features of an Emerging New Entity. Indian J. Urol..

[31] Bjornsson J., Short M.P., Kwiatkowski D.J., Henske E.P. (1996). Tuberous Sclerosis-Associated Renal Cell Carcinoma. Clinical, Pathological, and Genetic Features. Am. J. Pathol..

[32] Lam H.C., Nijmeh J., Henske E.P. (2016). New Developments in the Genetics and Pathogenesis of Tumours in Tuberous Sclerosis Complex. J. Pathol..

[33] Kucejova B., Peña-Llopis S., Yamasaki T., Sivanand S., Tran T.A.T., Alexander S., Wolff N.C., Lotan Y., Xie X.-J., Kabbani W. (2011). Interplay between PVHL and MTORC1 Pathways in Clear-Cell Renal Cell Carcinoma. Mol. Cancer Res..

[34] Caliò A., Marletta S., Settanni G., Rizzo M., Gobbo S., Pedron S., Stefanizzi L., Munari E., Brunelli M., Marcolini L. (2023). MTOR Eosinophilic Renal Cell Carcinoma: A Distinctive Tumor Characterized by MTOR Mutation, Loss of Chromosome 1, Cathepsin-K Expression, and Response to Target Therapy. Virchows Arch..

[35] Tjota M.Y., Chen H., Parilla M., Pankhuri W., Segal J.P., Antic T. (2020). Eosinophilic Renal Cell Tumors with a TSC and MTOR Gene Mutations Are Morphologically and Immunohistochemically Heterogenous. Am. J. Surg. Pathol..

[36] Tong K., Hu Z. (2021). FOXI1 Expression in Chromophobe Renal Cell Carcinoma and Renal Oncocytoma: A Study of the Cancer Genome Atlas Transcriptome–Based Outlier Mining and Immunohistochemistry. Virchows Arch..

[37] Williamson S.R. (2019). Renal Cell Carcinomas with a Mesenchymal Stromal Component: What Do We Know so Far?. Pathology.

[38] Skala S.L., Wang X., Zhang Y., Mannan R., Wang L., Narayanan S.P., Vats P., Su F., Chen J., Cao X. (2020). Next-Generation RNA Sequencing–Based Biomarker Characterization of Chromophobe Renal Cell Carcinoma and Related Oncocytic Neoplasms. Eur. Urol..

[39] Lam H.C., Siroky B.J., Henske E.P. (2018). Renal Disease in Tuberous Sclerosis Complex: Pathogenesis and Therapy. Nat. Rev. Nephrol..

[40] Pivovarcikova K., Alaghehbandan R., Vanecek T., Ohashi R., Pitra T., Hes O. (2022). TSC/MTOR Pathway Mutation Associated Eosinophilic/Oncocytic Renal Neoplasms: A Heterogeneous Group of Tumors with Distinct Morphology, Immunohistochemical Profile, and Similar Genetic Background. Biomedicines.

[41] Kapur P., Brugarolas J., Trpkov K. (2023). Recent Advances in Renal Tumors with TSC/mTOR Pathway Abnormalities in Patients with Tuberous Sclerosis Complex and in the Sporadic Setting. Cancers.

[42] Guo Q., Yao X., Yang B., Qi L., Wang F., Guo Y., Liu Y., Cao Z., Wang Y., Wang J. (2024). Eosinophilic Solid and Cystic Renal Cell Carcinoma: Morphologic and Immunohistochemical Study of 18 Cases and Review of the Literature. Arch. Pathol. Lab. Med..

[43] McKenney J.K., Przybycin C.G., Trpkov K., Magi-Galluzzi C. (2018). Eosinophilic Solid and Cystic Renal Cell Carcinomas Have Metastatic Potential. Histopathology.

